# Nemertide Alpha-1 as a Biopesticide: Aphid Deterrence, Antimicrobial Activity, and Safety Aspects

**DOI:** 10.3390/md23100388

**Published:** 2025-09-29

**Authors:** Quentin Laborde, Katarzyna Dancewicz, Erik Jacobsson, Adam A. Strömstedt, Taj Muhammad, Camilla Eriksson, Blazej Slazak, Ulf Göransson, Håkan S. Andersson

**Affiliations:** 1 Pharmacognosy, Department of Pharmaceutical Bioscience, Faculty of Pharmacy, Uppsala University, 751 23 Uppsala, Sweden; 2 Department of Botany and Ecology, University of Zielona Góra, Szafrana 1, 65-516 Zielona Góra, Poland; 3 W. Szafer Institute of Botany, Polish Academy of Sciences, 46 Lubicz St., 31-512 Cracow, Poland; 4 Department of Medical Biochemistry and Biophysics, Karolinska Institutet, 171 77 Stockholm, Sweden

**Keywords:** biopesticide, peptide toxin, pest control, repellent, Nemertea, aphid, *Myzus persicae*

## Abstract

Aphid control often relies on synthetic pesticides, but their overuse has raised concerns about resistance development and negative impact on wildlife and human health. Consequently, the search for new biopesticide agents has gained significant attention. Nemertide alpha-1, a peptide toxin from the marine nemertean worm *Lineus longissimus* (Gunnerus, 1770), is known for its pesticide activity but has less documented biological safety. This study investigates the aphid feeding deterrence and biological safety of the experimental biopesticide nemertide alpha-1. Nemertide alpha-1 demonstrated a clear dose-dependent repellent effect on the penetration behaviour of the green peach aphid (*Myzus persicae*, Sulzer). It also demonstrates bacteriostatic and bactericidal effects in an MIC (Minimum Inhibitory Concentration) assay, respectively, on *E. coli* (MIC: 112.5 µM) and *S. aureus* (MIC: 28.4 µM). In a bacterial liposome leakage assay, nemertide alpha-1 exhibits a less pronounced effect than the melittin control (20% maximum leakage at 100 µM), strengthening the hypothesis on the specificity of its neurotoxic mode of action. It is not toxic to mammalian cell U-937 GTB with only a slight decline in the percentage of survival at the highest concentration tested (80 µM). Finally, nemertide alpha-1 displays thermal stability over time for four weeks in three different conditions: cold (6 °C), room temperature (20–24 °C), and physiological temperature (37 °C). Nemertide alpha-1 deters green peach aphid feeding in the low micromolar range and exhibits low antimicrobial properties and very low toxicity to human cells. Its potential utility is further underscored by thermal stability over time.

## 1. Introduction

Aphids are significant pest insects, primarily due to their feeding on plant phloem and transmitting viral diseases [1]. Traditional strategies to combat aphids and other insects involve low-molecular-weight synthetic pesticides (e.g., organochlorines, organophosphates, carbamates, and pyrethroids). While synthetic pesticides offer immediate and effective action, their overuse leads to health risks and resistance development [2,3]. For these reasons, the Integrated Pest Management (IPM) concept was established at the end of the 1950s, recommending the use of a combination of cultural, genetic, biological, and behavioural strategies [4]. To prevent resistance development, it is advised to use pesticides in combination and rotate their use frequently. In parallel, there is a push to replace conventional pesticides with biopesticides for safer food consumption [5]. The negative effects of chemical pesticides regularly force new restrictions, highlighting the urgent need for biopesticide development in crop protection [6].

Animal toxins represent a rich source of biologically active compounds. Present in venoms and poisons, these toxins are utilized by a wide range of species to immobilise or eliminate both predators and prey. They typically comprise complex mixtures of small molecules, peptides, and proteins; some of which are currently being investigated for therapeutic and agricultural applications [7,8,9]. The search for novel peptide-based biopesticides has accelerated with advances in mass spectrometry and -omics technologies, leading to the identification of several promising candidates [10,11,12,13]. This progress has enabled more efficient study of previously underexplored phyla, such as Nemertea [14]. Most of the just over 1300 nemertean species thus far identified are predatory, and toxins have been shown to play a crucial role in both prey capture and defence. Among these, the recently discovered nemertide alpha-1, isolated from the epidermal mucus of the ribbon worm *Lineus longissimus* (Gunnerus, 1770), has demonstrated potent and selective biopesticide activity in vitro [15]. Nemertide alpha-1 is a 31 amino acid residue inhibitory cysteine-knot (ICK) peptide (Figures S1 and S2) of 3309 Da (Table S1) acting on voltage-gated sodium channels (VGSCs) and preventing their fast inactivation, a mechanism that distinguishes it from most other biopesticide candidates. The EC_50_ on the voltage-gated sodium channel of the German cockroach (BgNa_v_1 of *Blatella germanica* L.) is 8.6 nM, with a 100-fold decrease in activity observed on mammalian Na_v_ channels [15]. Complete inhibition of inactivation was also observed for the sodium channels of *Drosophila melanogaster* (Meigen, 1830) and *Varroa destructor* (Andersson & Trueman, 2000) Na_v_ channels at 1 µM [15]. Furthermore, the insecticidal activity of a recombinant nemertide alpha-1 propeptide has been reported in adult aphids (*Acyrthosiphon pisum*, *Harris*, 1776, and *M. persicae*, *Sulzer*, 1776), cabbage moth larvae (*Mamestra brassicae*, *Linnaeus*, 1758) *and adult bees* (*Apis mellifera*, *Linnaeus*, 1761) [16]. As a mucosal toxin, nemertide alpha-1 is a poisonous component of the mucus secreted by *L. longissimus*, rather than a constituent of a venom system, and it is believed to have evolved for feeding deterrence; to exert oral toxicity against organisms that ingest it. Given these promising findings, we sought to further investigate the insecticidal potency of nemertide alpha-1 from both agricultural and human health perspectives. We hypothesize that nemertide alpha-1 functions as a selective biopesticide with minimal off-target effects on non-target organisms, including mammals and beneficial insects. The objective of this study is to evaluate its biological activity in three distinct contexts: (i) feeding deterrence on green peach aphids (*Myzus persicae*), (ii) cytotoxicity on mammalian cells, and (iii) antimicrobial and membrane-disruptive effects on bacterial cells.

## 2. Results

### 2.1. Antimicrobial Activity and Liposome Leakage Assay

Nemertide alpha-1 and benchmark antimicrobial peptide (AMP) (LL-37) were subjected to antibacterial testing using the two-step microdilution assay, which is designed for testing AMPs without any activity-inhibiting constituents present. For *Staphylococcus aureus*, nemertide alpha-1 showed moderate activity, with a MIC (Minimum Inhibitory Concentration) of 28.35 μM. At the MIC, the peptide exhibited bactericidal activity, completely killing the bacteria, as confirmed by the agar plating results. However, against *Escherichia coli*, the MIC was 112.5 μM. At this concentration, the peptide was bacteriostatic, meaning it only inhibited the growth of the bacteria but did not kill them. LL-37 showed low MIC against *S. aureus* and *E. coli*, 1.25 and 1.875 µM, respectively.

**Figure 1:**
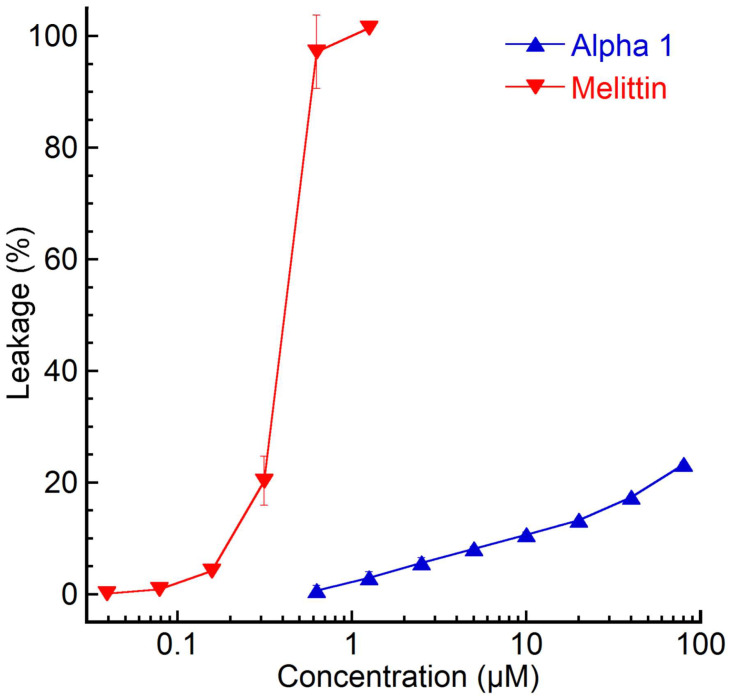
Total percentage of liposome permeability after 45 min. Results shown represent the mean from triplicate experiments with standard deviations and are presented as per cent of total leakage generated with Triton X-100 and subtraction of the baseline value The EC_50_ value for melittin (0.38 µM) was calculated using a sigmoidal dose–response curve fitting, with the leakage percentage (0–100 constraints) as a function of the peptide concentration (log10).

Nemertide alpha-1 was also tested in a bacterial liposome leakage assay where it was incubated with liposomes for 45 min. It demonstrated a maximum of 20% leakage at the highest concentration (100 µM) and no dose–response behaviour, while the positive control melittin exhibited a dose–response behaviour with an EC_50_ of 0.38 µM after 45 min incubation (Figure 1).

### 2.2. Cytotoxic Activity

**Figure 2:**
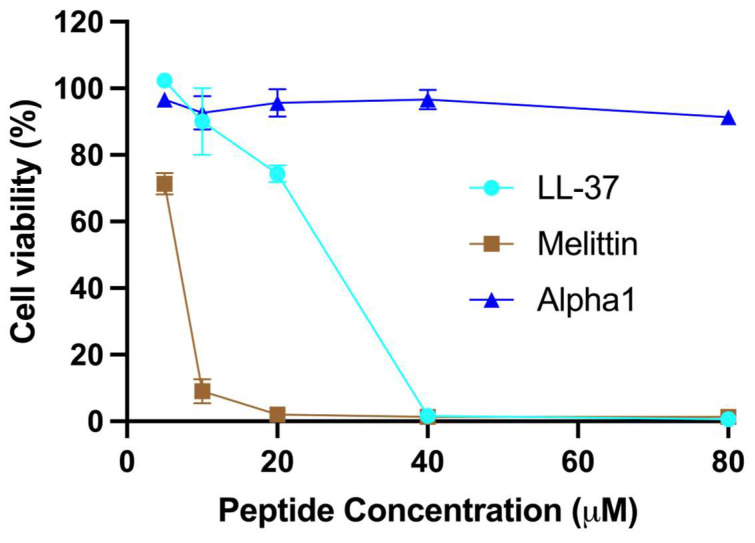
The fluorometric microculture cytotoxicity assay was used to generate data on lymphoma cell toxicity for nemertide alpha-1 peptide. This assay relies on cellular esterases, which, when the cell’s plasma membranes remain intact, hydrolyse the fluorescein diacetate (FDA) probe. Results showed that the nemertide alpha-1 peptide led to only a slight decrease in cell viability even at higher concentrations, while the control peptides LL-37 and melittin caused cell lysis at low concentrations.

The cytotoxic nature of certain AMPs is mainly attributed to their non-specific binding to negatively charged membranes, thus necessitating thorough testing for potential crop plant protection or therapeutic applications [17]. In the present study, a fluorometric microculture cytotoxicity assay (FMCA) was utilized to assess the cytotoxicity of this peptide on a human lymphoma cell line (U-937 GTB). Overall, nemertide alpha-1 demonstrated markedly higher cell viability as compared to LL-37 and melittin. As expected from its presence in bee venom, melittin shows the most pronounced concentration-dependent decrease in cell viability. Following melittin, LL-37, a human endogenous AMP, showed significant toxicity. Nemertide alpha-1 exhibited minimal or no effect on cell viability, except at the highest concentration tested (80 µM), where a slight decline in viability was observed (Figure 2).

### 2.3. Effects on Aphids’ Feeding Behaviour

**Figure 3:**
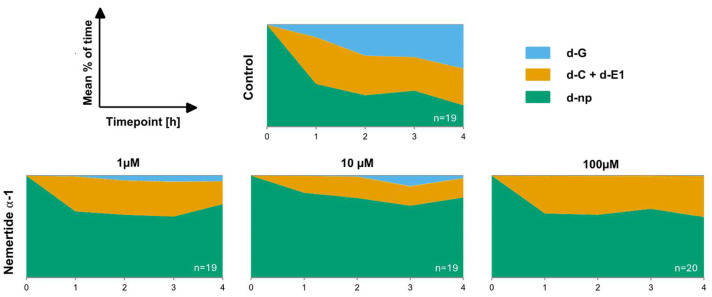
Sequential changes in the probing behaviour of *M. persicae* on sucrose diets with different concentrations of nemertide α-1 (1 µM, 10 µM, and 100 µM) during 4-h EPG experiments. The mean proportion of time spent on different stylet activities was measured: pathway (d-C), salivation into the diet (d-E1), active ingestion of the diet (d-G), non-penetration—aphid stylets remaining outside of the diet (d-np). Measurements for all individual aphids were included in the analysis. Values represent the means of n replicates per treatment (n = 19: control, n = 19: 1 µM, n = 19: 10 µM and n = 20: 100 µM).

The sequential variation in feeding behaviours was monitored on a control diet and diets containing concentrations of nemertide alpha-1 (1 µM, 10 µM, and 100 µM) through the four-hour recording (Figure 3). When the experiment started all aphids exhibited similar non-probing activity. The mean proportion of time spent on different stylet activities was measured: non-penetration—aphid stylets remaining outside of the diet (d-np); pathway (d-C) and salivation into the diet (d-E1); active ingestion of the diet (d-G). Nemertide alpha-1 had a strong feeding deterrent effect across all tested concentrations, leading to a higher proportion of the non-penetrating phase (d-np) throughout the experiment compared to the control. Within the first hour, 65% of individuals at 1 µM, 83% at 10 µM, and 63% at 100 µM kept their stylets outside the diet (d-np), whereas only 42% did so in the control group. The proportion of the active ingestion phase (d-G) gradually increased to 43% after 4 h for the control diet, whereas ingestion activity barely increased for the nemertide alpha-1 diets. For the 1 µM nemertide alpha-1 diet, a maximum of 6% ingestion activity was observed after 3 h, on the 10 µM nemertide alpha-1 diet a maximum of 10% ingestion activity was observed after, 3 h and on 100 µM nemertide alpha-1 diet a maximum of 0.4% ingestion activity was observed after 4 h. Ingestion from the nemertide alpha-1 diet was significantly lower than from the control diet by the end of the experiment. Consumption rates were 5% at 1 µM, 2% at 10 µM, and only 0.4% at 100 µM, compared to 43% for the control diet. Conversely, non-probing activity remained stable for the nemertide alpha-1 diets with values between 60–70% for 1 µM nemertide alpha-1 diet, 70–80% for 10 µM nemertide alpha-1 diet, and 60–70% for 100 µM nemertide alpha-1 diet. The corresponding value of the control was 21% after 4 h (Figure 3).

**Figure 4:**
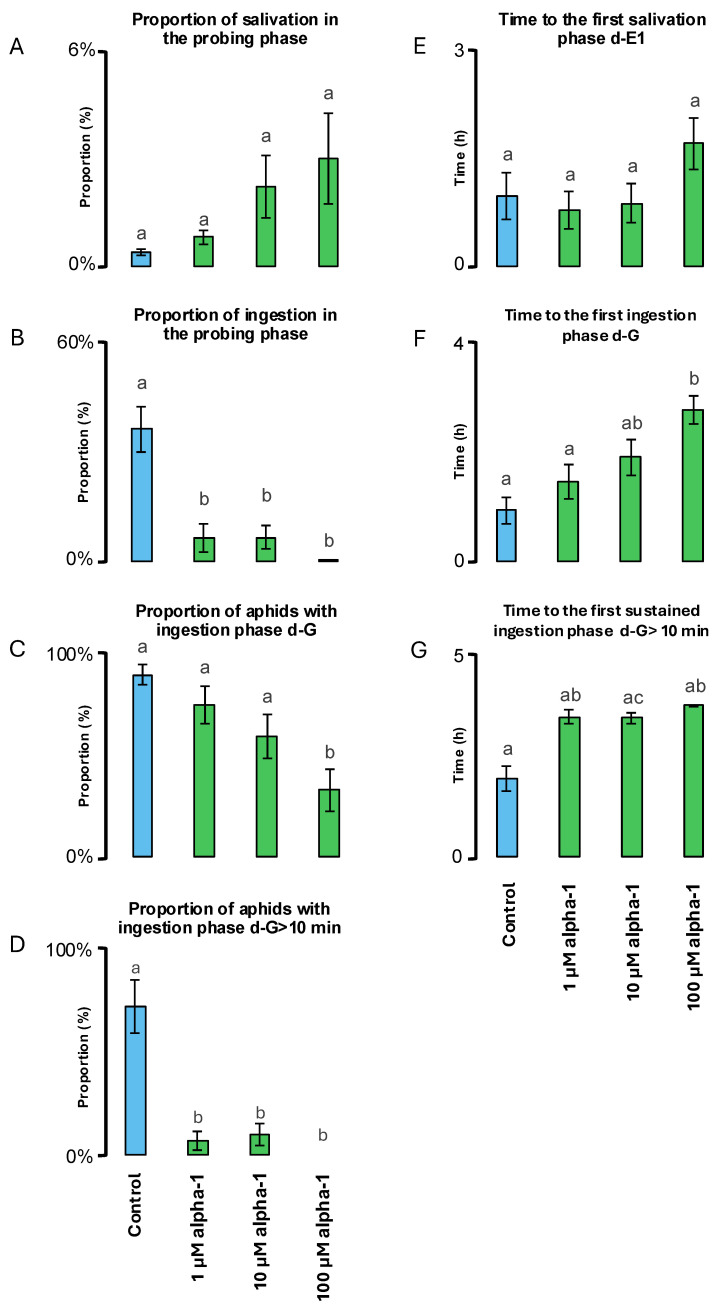
Behavioural effects of nemertide α-1 on *M. persicae* during 4-h EPG experiments. (**A**), the proportion of salivation in the probing phase (d-E1/d-C + d-E1 + d-G). (**B**), the proportion of the ingestion phase in the probing phase (d-G/d-C + d-E1 + d-G). (**C**), proportion of aphids with ingestion phase (d-G). (**D**), the proportion of aphids with sustained ingestion phase (d-G > 10 min). (**E**), time from 1st probe to 1st salivation phase (d-E1). (**F**), time from 1st probe to 1st ingestion phase (d-G). (**G**), time from 1st probe to 1st sustained ingestion phase (d-G > 10 min). Measurements for all individual aphids were included in the analysis. Values represent the means of n replicates and bars indicate standard errors (n = 19—control, n = 20—1 µM, n = 19—10 µM, and n = 20—100 µM). Different letters indicate statistically significant differences with the control (the letter ‘a’) and between groups of aphids on diets containing various concentrations of nemertide α-1 (letter ‘b’, at *p* < 0.05).

Further detailed analyses provided information on the statistical significance and dose dependence of the nemertide alpha-1 diets vs. the control diet (Table 1). The total time of non-probing activity was significantly longer (2–2.5 times) for all nemertide alpha-1 diets compared to the control diets. Accordingly, the nemertide alpha-1 diets also demonstrated significantly shorter (2–3 times) total time of probing phase compared to the control diet. The proportion of salivation in all aphid activities in the diets (d-E1/d-C + d-E1 + d-G) and total time of the salivation phase was not significantly different between the control diet and the nemertide alpha-1 diets (Table 1, Figure 4A). The total duration of the ingestion phase was significantly shorter for all nemertide alpha-1 diets with for the 100 µM nemertide alpha-1 diet being 179 times shorter (0.4 ± 0.2 min) compared to the control diet (71.6 ± 14.2 min) (Table 1, Figure 4B). The number of probes was slightly affected by the treatments with the 10 µM nemertide alpha-1 diet (18.6 ± 3.0) being the only treatment significantly different from the control diet (36 ± 5.2). The proportion of aphids with an ingestion phase was significantly lower on the 100 µM nemertide alpha-1 diet than on the control diet (Table 1, Figure 4C). Similarly, the proportion of aphids with sustained ingestion phase (>10 min) was significantly lower on nemertide alpha-1 diets compared to the control diet (Figure 4D). The number of sustained ingestion phases was 12 and 6 times lower on nemertide alpha-1 (1 µM, 10 µM, respectively) diets than on control diet. Aphid-sustained ingestion was completely inhibited after application of the 100 µM nemertide alpha-1 diet (no aphid showed d-G > 10 min) (Table 1). No significant differences between nemertide alpha-1 and control diets were observed concerning time from the first probe to the first salivation phase d-E1 (Figure 4E). However, 10 µM and 100 µM nemertide alpha-1 diets exhibited significantly higher time (2–3 times) from the first probe to first ingestion phase d-G compared to 1µM nemertide alpha-1 and control diets (Figure 4F). Finally, the time from first probe to first sustained ingestion phase d-G > 10 min was significantly higher (2 times) for the nemertide alpha-1 diets when compared to the control diet (Figure 4G).

### 2.4. Temperature Stability

**Figure 5:**
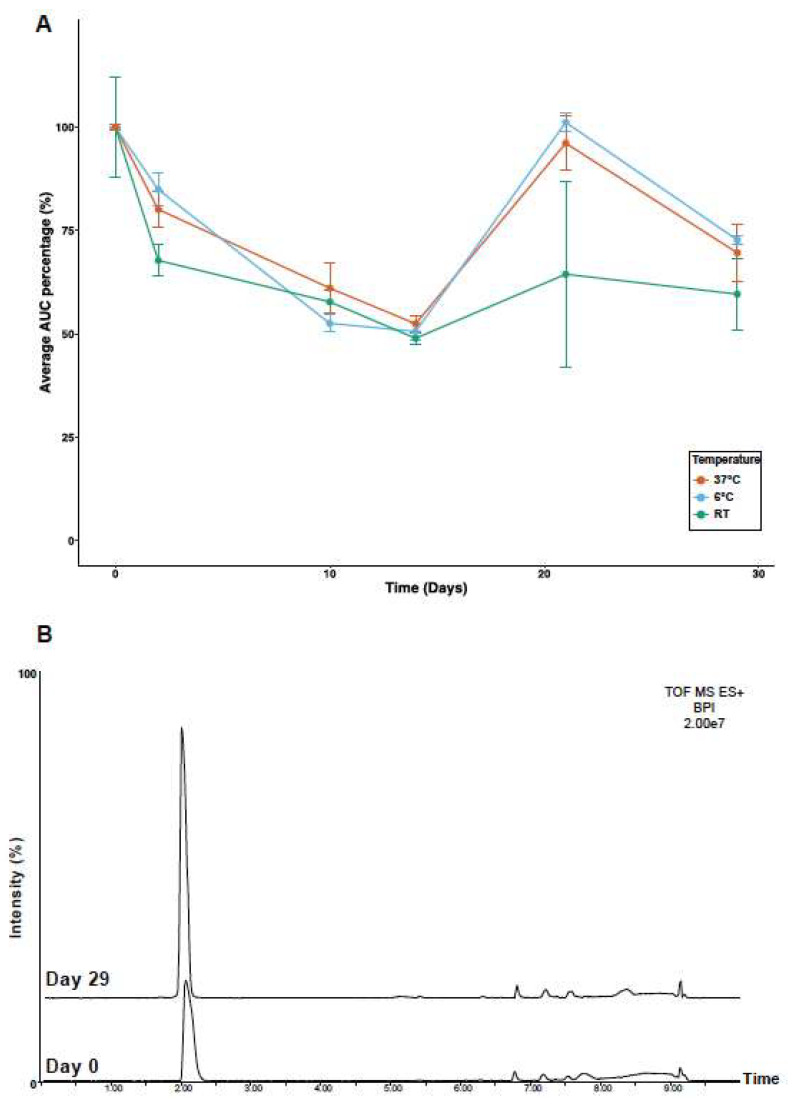
Temperature stability evaluation over 30 days in three temperature conditions. (**A**), Line plot of the normalized average area under the curve (AUC) percentage from alpha-1 temperature stability evaluation over 30 days. Blue: cold (6 °C), green: room temperature (20–24 °C, RT), and red: physiological temperature (37 °C). All measurements were performed in triplicate and data points are shown as average percentages with standard deviation. (**B**), Base peak intensity (BPI) chromatograms of nemertide alpha-1 at 0 days and 29 days for the physiological condition (37 °C).

The stability of nemertide alpha-1 was assayed using LC-MS over time and at different temperatures. Mass spectra in a range of 100–2000 *m*/*z* were acquired to monitor possible degradation products. However, only ions corresponding to the full-length peptide were detected at all time points and temperatures, demonstrating stability over 30 days. Quantities were assayed by AUC (Area Under the Curve) of 828.1034 (4 z) and 425.2147 (1 z), after removing an aliquot from all samples followed by immediate analyses on that day. Results show a decline from day 0 to day 2 to 80–70% of their initial AUC, which then declined again to 50% up to day 14 (Figure 5A). Later time points gave increasing values; this effect we attributed to evaporation effects of the small volume of samples available for analyses. The total absence of fragments indicates that the initial loss is explained by the absorption of peptides into the tubes. Finally, no degradation products were detected through time for the highest temperature condition (Figure 5B). Overall, results show that nemertide alpha-1 is a stable peptide over the period used for the assays above.

## 3. Discussion

### 3.1. Nemertide Alpha-1 Membrane Interactions

Although substantial data describes the toxicity of nemertide alpha-1 in vitro, and in vivo in crustaceans and cockroaches [15] knowledge is scarce concerning the effects of nemertide alpha-1 on bacterial and human cells. No cell-lytic effects had yet been observed in previous studies, so the absence of lytic effects obtained in the present study confirmed our initial expectations. AMPs often act by targeting and disrupting bacterial membranes, leading to cell lysis. To explore whether our peptide follows a similar mechanism, we first determined its MIC values against representative bacterial strains, as a measure of antimicrobial potency. To assess whether this activity correlates with membrane disruption, we performed liposome leakage assays using synthetic bacterial membrane models. Finally, since membrane-targeting peptides can also affect mammalian cells, we evaluated cytotoxicity to assess the therapeutic window and selectivity of our compound.

### 3.2. Antibacterial Potency

Nemertide alpha-1 antimicrobial activity on *S. aureus* (MIC: 28.4 µM) was substantially lower compared to known AMPs such as LL-37 (MIC: 0.67 µM), KR-12 (MIC: 10 µM) and gomesin (MIC: 1.6–3.15 µM) [18,19,20]. This was also true for nemertide alpha-1 antimicrobial activity on *E. coli* (MIC: 112.5 µM) as compared again to known AMPs such as LL-37 (MIC: 40 µM), KR-12 (MIC: 2.5 µM) and gomesin (MIC: 0.32–3.4 µM) [19,20,21]. Interestingly, nemertide alpha-1 was more potent against Gram-positive bacteria than Gram-negative bacteria. This could be explained by the fact that nemertide alpha-1 did not display a pore-forming mechanism in contrast to most AMPs. Pore-formation is usually attributed to non-specific binding to negatively charged membranes [22]. Nemertide alpha-1 has a net positive charge of +3 at physiological pH which is lower compared to most AMPs (+3 to +9) which exhibit excesses of basic residues over neutral polar and acidic residues [23]. However, at this point, the assessment of nemertide alpha-1 antimicrobial activity was only preliminary, as it was performed on only two strains of bacteria.

### 3.3. Membrane Disruption and Cell Toxicity

Nemertide alpha-1 demonstrated low membrane-lytic effects on liposomes at tested concentrations. These observations are in agreement with the low antimicrobial activity described above. Indeed, nemertide alpha-1 exhibited markedly lower activity than the benchmark melittin which is positively charged at physiological pH and penetrates cell membranes [24,25,26]. Nemertide alpha-1 also showed very low human cell cytotoxicity in U-937 GTB cells, exhibiting high survivability when exposed to the high concentration of nemertide alpha-1. These cells are considered robust and have been validated as cellular models for screening modulatory agents [27]. The FMC assay relies on cellular esterases, which, when the cell’s plasma membranes remain intact, hydrolyse the fluorescein diacetate probe. U-937 cell survivability results featured supplementary evidence on the very low membranolytic effect of nemertide alpha-1 on bacterial and mammalian cells. This suggested that a different mechanism of action underlies the antimicrobial activity of nemertide alpha-1 on Gram-positive bacteria. VGSCs are also present in bacteria under the name NaChBac, which constitutes a superfamily of VGICs [28,29,30,31,32]. NaChBacs comprise one six-transmembrane segment instead of the four transmembrane segments present in VGSCs from pluricellular organisms [32]. Hence, a potential explanation could be that nemertide alpha-1 exerts a similar toxicity mechanism on bacterial NaChBacs and vertebrate/invertebrate VGSCs by binding to one of the extracellular loops of the respective channels.

### 3.4. Nemertide Alpha-1 Feeding Deterrence

The use of diets containing nemertide alpha-1 affects the feeding behaviour of *M. persicae* aphids. The three tested concentrations (1, 10, and 100 µM) significantly increased the time proportion and duration of the non-probing phase. It also reduced the time proportion of both the probing phase and the ingestion phase with a significantly lower duration of the nutrient uptake during the ingestion phase. Surprisingly, the 10 µM nemertide alpha-1 diet exhibited a stronger deterrent effect than the 100 µM diet during the early stages of the feeding process, specifically the non-penetration and penetration phases. It also resulted in a greater reduction in the number of probes compared to the 100 µM concentration. This pattern deviates from the expected monotonically increasing dose–response curve. However, during the salivation and ingestion phases, the data did follow the anticipated dose-dependent trend. The stronger deterrent effect observed at the intermediate concentration may be explained by a saturation of gustatory receptors, where mid-level doses elicit the strongest response, while higher concentrations lead to receptor desensitisation or saturation. This response pattern may reflect a biphasic or hormetic effect, in which low doses stimulate, intermediate doses produce peak activity, and high doses diminish the effect due to toxicity, receptor saturation, or adaptive mechanisms. Another interesting observation was the increase in the duration of the salivation phase with the 100 µM nemertide alpha-1 diet despite not being significantly different from the other diets. Additionally, the 100 µM nemertide alpha-1 diet was the only diet exhibiting a significantly lower percentage of aphids with an ingestion phase longer than 10 min. From these results, it appears clear that nemertide alpha-1 provokes a deterrent effect on the feeding behaviour of aphids. This phenomenon was previously observed with the use of cyclotides (cyO2, cyO3, cyO13, and cyO19), cyclic polypeptides involved in plant defence, from *Viola* sp. [33]. It was hypothesised that the presence of peptides could partially influence aphids’ taste when interacting with gustatory organs [33]. Similarly to cyclotides, nemertide alpha-1 may also interact with insect feeding at the pre-digestive level. The hypothesis that nemertide alpha-1 invokes a defence mechanism, correlates well with the outcome of *M. persicae* recordings. Similarly to cyclotides, nemertide alpha-1 deterrent ability could be related to its ecological role in *L. longissimus* and other *Lineidae* species when they are subjected to predators. Indeed, insects and crustaceans share the same phylum (Arthropoda) and even the same clade (Pancrustacea) explaining morphological and physiological similarities [34]. The deterrent effect on feeding behaviour from nemertide alpha-1 is in agreement with the results from [16] who evaluated a recombinant nemertide alpha-1 propeptide with regard to its potential as a biopesticide candidate. Repellent effect on feeding behaviour and acute toxicity are compelling characteristics when developing a biopesticide formulation for plant protection.

### 3.5. Nemertide Alpha-1 Thermal Stability

We also previously demonstrated that nemertide alpha-1 was amenable to protease degradation (trypsin, chymotrypsin, endoproteinase Glu-C) after reduction and alkylation in conjunction with tandem mass spectrometry experiments [15]. Therefore, in the presence of a naturally reducing reagent nemertide alpha-1 can be expected to exhibit increased sensitivity to protease degradation. This sensitivity to enzymatic degradation suggests that the risk for bioaccumulation and bio amplification in the food chain should be negligible. The additional data on temperature stability suggests stability in the range of 60–70% for nemertide alpha-1 through a period of one month, granted that certain variations were observed in this experiment. AUC was normalized versus a solvent peak to adjust for variations in instrument sensitivity over time. Further variation could potentially be explained by solvent evaporation [35]. Finally, mass analyses did not demonstrate any significant alteration of the peptide purity over time, regardless of temperature, suggesting that temperature is not an issue for nemertide alpha-1.

### 3.6. Limitations and Future Perspectives

In the present paper, we have investigated feeding deterrence, where bioavailability per se is less relevant. However, if making use of the neurotoxicity of nemertide alpha-1 is the goal, indications exist that bioavailability may be a limiting factor. Previous work has demonstrated the toxicity of nemertide alpha-1 when injected into the green shore crab (*Carcinus maenas*), the Dubia cockroach (*Blaptica dubia*), and *adult bees (Apis mellifera)* [15,16] but this method is not useful for large-scale administration of biopesticides. Several strategies are available to circumvent bioavailability challenges. Chemical modification of the side chains and cyclisation can improve peptide stability, protecting them against peptidases [36,37,38]. The use of poly-lysine or bioconjugate peptides can enhance cellular uptake [39,40]. Peptides can also be combined with cell-penetrating peptides (CPP) increasing cellular uptake [41,42]. Another possibility would be to engineer plant systems to synthesise higher quantities of the peptide of interest in their cellular tissues [43,44,45]. The low cytotoxicity of nemertide alpha-1 towards human cells suggests a favourable safety profile, positioning it as a promising alternative to conventional neurotoxic pesticides, which often exhibit harmful effects on non-target organisms and pose health risks to humans. This selective toxicity is particularly valuable in developing next-generation pest control agents with reduced environmental and health impact. To further assess the ecological safety of nemertide alpha-1, future studies will include testing its effects on non-target organisms, including beneficial insects such as pollinators (e.g., bees), to better understand its potential selectivity and environmental compatibility. Another aspect is that the peptide used in the present study was chemically synthesized [15]. However, from both scalability and environmental sustainability perspectives, heterologous expression in microbial or plant-based systems (e.g., bacteria, yeast, or plants) [46] would be a more favourable alternative.

## 4. Materials and Methods

### 4.1. Peptide Synthesis and Quality Assessment

Nemertide alpha-1 was synthesized using FMOC-based solid phase peptide synthesis as described by Jacobsson et al. [15], and purity was 95%, as assessed in that paper. The peptide used in the present study came from the same batch as described by Jacobsson et al. [15]

### 4.2. Minimum Inhibitory Concentration (MIC) Assay

The antimicrobial activity of nemertide alpha-1 was evaluated using a two-step microdilution assay adapted for peptides by Strömstedt et al. [47]. The experiment involved two types of microorganisms: *Escherichia coli* ATCC 25922 (Gram-negative), and *Staphylococcus aureus* ATCC 29213 (Gram-positive), which were used for minimum inhibitor concentration determination (MIC). The bacterial strains were obtained from the Department of Clinical Bacteriology at Lund University Hospital, Sweden. The microbial strains were grown overnight in 3% tryptic soy broth (TSB) at 37 °C until the mid-logarithmic phase. The microbial suspension was subsequently washed twice with a 10 mM Tris buffer (set to pH 7.4 at 37 °C).

A 2-fold serial dilution of the peptides in a Tris buffer using 96-well plates (U-shaped, untreated polystyrene) was prepared. The microbial density was measured at OD_600_ and then diluted in Tris buffer. Next, 50,000 cfu were added to each well, resulting in a final volume of 100 µL. After 1 h of aerobic incubation at 37 °C, 5 µL of 20% (*w*/*v*) TSB medium was added to each well (resulting in 1% TSB) and the plates were further incubated at 37 °C for 16–18 h. The MIC was defined as the lowest peptide concentration that fully inhibited visible bacterial growth by the indicated time. The resulting MIC values are median values from triplicate independent experiments. The deviations between independent experiments did not exceed one dilution factor. The bactericidal versus bacteriostatic activities of nemertide alpha-1 were assessed from samples of resuspended MIC well contents taken after the initial Tris buffer incubation step of the microdilution assay. The samples were diluted to 20% and plated on LB-agar, with an expected inoculum of approximately 1000 cfu per plate.

### 4.3. Bacterial Liposome Leakage Assay

Liposomes were prepared following the method described in Strömstedt et al. [48]. *Escherichia coli* polar lipid extract was used to create lipid films on round-bottom flask walls. The fhilms were then resuspended in a solution containing 100 mM 5(6)-carboxyfluorescein in Tris buffer at 55 °C. To reduce multilamellar structures and polydispersity, the suspensions were extruded multiple times through a 100 nm polycarbonate membrane. Un-trapped carboxyfluorescein was eliminated through gel filtration. To assess membrane permeability, carboxyfluorescein efflux from the liposomes to a low-concentration environment was measured. This caused a loss of self-quenching and increased the fluorescence signal. Nemertide alpha-1 was diluted (1.2 µM–80 µM) in Tris buffer and added to 96-well plates, along with controls (background) and Triton X-100 (maximum leakage). Liposome solution was then added to the plates, resulting in a final lipid concentration of 10 μM in 200 µL. The effects of nemertide alpha-1 concentration on the liposomes were monitored for 45 min, after which the initial leakage subsided.

Results, presented as percentages of total leakage relative to Triton X-100 and baseline subtraction, represent the average of three triplicate experiments with standard deviations. The EC_50_-values, representing 50% of total leakage, were calculated using a sigmoidal dose–response curve for leakage percentage (0–100 constraints) as a function of sample concentration (log10). Melittin, a cytolytic peptide from bee venom, was used as a benchmark for comparison (3 nM–1.2 µM). The melittin used in the study was purified from the apitoxin of apiary Carniolan hybrid honeybees (*Apis mellifera* L.) collected by the Department of Bee Research, Plant Protection Research Institute, Agricultural Research Centre, Egypt.

### 4.4. Cytotoxicity Assay

Cytotoxicity of the peptides was assessed on the human lymphoma cell line U-937 GTB (Department of Immunology, Pathology and Genetics, Uppsala University, Uppsala, Sweden) [49] using a fluorometric microculture cytotoxicity assay (FMCA), as described previously by Lindhagen et al. [50]. The cell line was obtained as described earlier, was maintained in RPMI 1640 complete medium (Sigma–Aldrich, St. Louis, MO, USA) supplemented with 10 % heat-inactivated foetal bovine serum, 2 mM glutamine, 50 μg/mL streptomycin, and 60 μg/mL penicillin (all from Hyclone, Cramlington, UK), and kept under standard incubation conditions (humidified atmosphere of 37  °C, 5 % CO_2_). The cultures were monitored and passaged twice weekly and were harvested during the exponential growth phase. For the FMCA, peptide 2-fold serial dilutions were prepared in 96-well plates, followed by the addition of 20,000 cells in fresh growth medium to each well (to a total volume of 200 μL). The plates were incubated for 72 h under standard incubation conditions.

Once the incubation time had elapsed, the plates were centrifuged at 100× *g* for 5 min at 37 °C, the medium was removed, and the cells were washed with PBS (Phosphate-Buffer Saline). Next, 100 μL fluorescein diacetate (FDA) (10 μg/mL in physiological buffer) was added to each well. After incubation for 40 min, the fluorescence was measured at 485 nm excitation, and 538 emissions using a Varioskan Flash plate reader (Thermo Scientific, Waltham, MA, USA). The fluorescence intensity in each well was proportional to the number of living cells, and the cytotoxic activity was inversely proportional to the fluorescence intensity. The activity of the peptide was reported in terms of a survival index (SI), which was defined in terms of the fluorescence in the experimental wells expressed as a percentage of that in the control wells after the fluorescence of the blanks had been subtracted.

### 4.5. Aphid EPG Measurements

#### 4.5.1. EPG Principle

Experimental work was performed at the Department of Botany and Ecology at the University of Zielona Góra (Poland) using a previously developed method from Dancewicz et al. [33]. The method used was the electrical penetration graph (EPG) technique [51,52,53]. In short, a fine gold wire is glued to the back of an insect, and another wire is connected to the food source, which is usually a plant. Electrical current is run through the circuit, and voltage fluctuations can be recorded when the insect probes, salivates, and ingests food with its stylet through the plant tissues. The plant-insect relationship is thus converted to an electrical signal which can be monitored and recorded over time.

#### 4.5.2. *M. persicae* Rearing Conditions Before EPG Recording

For the EPG recordings, aphid apterous females or nymphs of *M. persicae* were used. These were obtained from a stock culture kept at the University of Zielona Góra, Poland. The stock culture was maintained on Chinese cabbage (*Brassica rapa* subsp. *pekinensis*, Lour., Hanelt) at 20 °C, 65% relative humidity (RH), and a photoperiod of 16 h light:8 h dark cycle.

#### 4.5.3. Preparation of Sucrose Diets for EPG Recording

The basic diet used in all experiments was a 20% sucrose solution chosen because of its highly phagostimulatory effect towards *M. persicae* [54,55,56]. Using a simple diet allowed the elimination of any effect of nutrients on aphid probing behaviour. Lyophilised amounts of nemertide alpha-1 were reconstituted in the sucrose solution to obtain final concentrations of: 1 μM, 10 μM and 100 μM. The control diet consisted of only 20% sucrose. A total of 0.5 µL liquid diet was added to each aphid feeding chamber (5 mm diameter, 1 mm height), which was covered with one layer of stretched Parafilm M^®^ (Merck & Co, Darmstadt, Germany; sterilized with 75% ethanol), following the method described by Sadeghi et al. [57]. Fresh diets were prepared directly before EPG recording.

#### 4.5.4. EPG Recording Conditions

Aphids were selected and dorsally tethered on the abdomen with a gold wire (1.5 cm long, 20 µm in diameter) and water-based conductive silver paint before the beginning of EPG recording. After the attachment of the wire electrode, the tethered aphids were starved for 1 h and then individually placed at the centre of each diet chamber. A second electrode was placed in the liquid diet. The probing behaviours of 77 aphid individuals per nemertide alpha-1 concentration were monitored for 4 h continuously. All experiments were performed in a Faraday cage in the laboratory (20 °C, 65% relative humidity, and a photoperiod of 16 h-light: 8 h-dark). Signals were recorded and analysed using the PROBE 3.1 software provided by Dr. W.F. Tjallingii (EPG-Systems; Dillenburg 12, 6703 CJ Wageningen, The Netherlands; www.epgsystems.eu). Two EPG rounds were usually recorded per day on both control (*n* = 19) and nemertide alpha-1 diets (1 µM: *n* = 19, 10 µM: *n* = 19, and 100 µM: *n* = 20) one after another.

#### 4.5.5. EPG Parameter Description

The following EPG patterns were distinguished from the sucrose diets: d-np (non-penetration—aphid stylets remained outside of the diet), d-C (pathway phase—penetration of the diet, including sheath salivation), d-E1 (salivation into the diet), d-G (active ingestion of the diet) previously found and identified in artificial diets [33,53,58,59]. The parameters derived from EPGs were analysed according to (1) duration (total duration of non-probing phase d-np; total duration of probing phase (d-C + d-E1 + d-G); total duration of salivation phase d-E1; total duration of ingestion phase d-G), (2) frequency (number of probes; number of probes with ingestion phase; number of ingestion phases d-G; number of sustained ingestion phases d-G > 10 min), (3) proportion (proportion of salivation in probing phase—d-E1/(d-C + d-E1 + d-G); proportion of ingestion phase in probing phase—d-G/(d-C+d-E1+d-G); proportion of aphids with ingestion phase d-G; proportion of aphids with sustained ingestion phase d-G > 10 min) and (4) sequence (time from first probe to first salivation phase d-E1; time from first probe to first ingestion phase d-G; time from first probe to first sustained ingestion phase d-G > 10 min).

#### 4.5.6. EPG Parameter Analysis

EPG parameters were calculated manually and individually for every aphid. The means and standard errors were subsequently calculated using an EPG analysis Excel worksheet created for this study. The results were analysed statistically using non-parametric Kruskal–Wallis’s test followed by post hoc Dunn’s multiple comparison tests in Statistica for Windows version 9.0 (StatSoft 2011). Separate comparisons were carried out for each group of aphids on a given nemertide alpha-1 diet. Each multiple comparisons included aphids on a given nemertide alpha-1 diet (1 µM, 10 µM, 100 µM), and control at *p* < 0.05 to show the dose-dependent effect of application of a given nemertide alpha-1 concentration on aphid behaviour.

#### 4.5.7. Temperature Stability

Nemertide alpha-1 stability was assessed on samples stored in Milli-Q water under three temperature conditions: cold (6 °C), room temperature (20–24 °C, RT), and physiological temperature (37 °C). Analytical runs were performed in triplicates for 30 days using an AQUITY Premier CSH C18 column (2.1 × 100 mm, 1.7 μm, 130 Å, Waters Corporation) on an AQUITY Premier UPLC system (Waters Corporation, Milford, MA, USA) coupled to a Xevo-G2-XS QToF mass spectrometer (Waters Corporation). A linear gradient from 5 to 95% acetonitrile with 0.1% formic acid at a flow rate of 0.65 mL/min over 10 min was used for analysis and the scan window was set to 100–2000 *m/z* on positive mode. Chromatograms were integrated using base peak intensity chromatograms and by selecting specific ions (828.1034, 4 z and 425.2147, 1 z) to obtain the area under the curve (AUC). The average AUC with standard deviation was plotted at each time point.

## 5. Conclusions

This study demonstrates that nemertide alpha-1 is a stable molecule, with several environmentally attractive features: it effectively deters green peach aphid feeding, shows low antimicrobial toxicity, and exhibits very low cytolytic and cell toxicity. These findings suggest that nemertide alpha-1 is unlikely to harm surrounding organisms. Its weak mammalian cytotoxicity also supports operator and food safety.

## Figures and Tables

**Table 1:** Probing behaviour of *M. persicae* (Sulz.) on sucrose diets with nemertide alpha-1 indicated with EPG parameters.

EPG Parameters	Total Duration of Non-Penetration Phase (h)	Total Duration of Penetration Phase ^†^ (h)	Total Duration of Salivation Phase ^‡^ (min)	Total Duration of Ingestion Phase ^§^ (min)	Number of Probes (#)	Number of Probes with Ingestion Phases (#)	Number of Ingestion Phases (#)	Number of Sustained Ingestion Phases ^¶^ (#)
Control n = 19	1.3 ± 0.2 ^a^	2.7 ± 0.2 ^a^	0.7 ± 0.2 ^a^	71.6 ± 14.2 ^a^	36.2 ± 5.2 ^a^	6.4 ± 1.2 ^a^	8.2± 1.3 ^a^	1.2 ± 0.2 ^a^
Nemertide alpha-1								
1 µM n = 19	2.58 ± 0.18 ^b^	1.4 ± 0.2 ^b^	0.7 ± 0.1 ^a^	9.9 ± 7.4 ^b^	41.4 ± 5.2 ^ac^	3.8 ± 1.5 ^ab^	4.5 ± 1.8 ^ab^	0.1 ± 0.07 ^b^
10 µM n = 19	3.12 ± 0.2 ^b^	0.9 ± 0.2 ^b^	0.8 ± 0.4 ^a^	8.3 ± 4.7 ^b^	18.6 ± 3.0 ^ab^	2.5 ± 0.6 ^a^	2.8 ± 0.8 ^b^	0.2 ± 0.09 ^b^
100 µM n = 20	2.5 ± 0.2 ^b^	1.5 ± 0.2 ^b^	4.1 ± 1.8 ^a^	0.4 ± 0.2 ^b^	44.8 ± 5.2 ^ab^	0.6 ± 0.2 ^b^	0.9 ± 0.3 ^b^	0.0 ± 0.0 ^b^
*p*	<0.001	<0.001	0.8256	<0.001	0.0016	<0.001	<0.001	<0.001

Values represent means ± SE, n = number of replicates, different letters in columns indicate statistically significant differences following the comparisons within groups consisting of control and different concentrations of a given nemertide alpha-1. The multiple comparisons refer to different concentrations of a given nemertide alpha-1 and control at *p* < 0.05 (Kruskal–Wallis test followed by posthoc Dunn’s multiple comparison test); ^†^ probing phase includes activities: pathway (d-C), salivation into the diet (d-E1), active ingestion of the diet (d-G); ^‡^ salivation into the diet (d-E1); ^§^ active ingestion of the diet (d-G); ^¶^ sustained ingestion of the diet (d-G > 10 min).

## Data Availability

The data presented in this study are available on request from the corresponding author.

## Supplementary Materials

The following supporting information can be downloaded at: https://www.mdpi.com/article/10.3390/md23100388/s1, Figure S1. Nemertide alpha-1 amino acids sequence; Figure S2. Nemertide alpha-1 3D ribbon structure with disulfide bonds coloured in yellow. PDB ID: 6ENA; Table S1. Molecular weight (g/mol) and monoisotopic mass (Da) of nemertide alpha-1.
