# Supplementary material for: Nemertide Alpha-1 as a Biopesticide: Aphid Deterrence, Antimicrobial Activity, and Safety Aspects
Source: Mar Drugs. 2025 Sep 29;23(10):388. doi: 10.3390/md23100388 (PMC12565628; doi:10.3390/md23100388)
Supplement: Supplementary file 1 [file marinedrugs-23-00388-s001.zip › marinedrugs-3885019-supplementary.pdf]

Supplementary Material

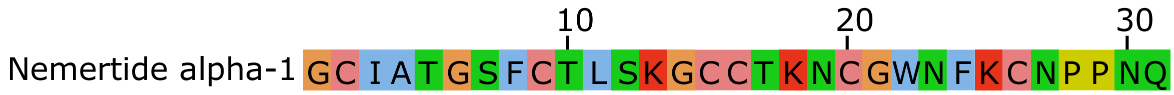

Figure S1. Nemertide alpha-1 amino acids sequence. [14].

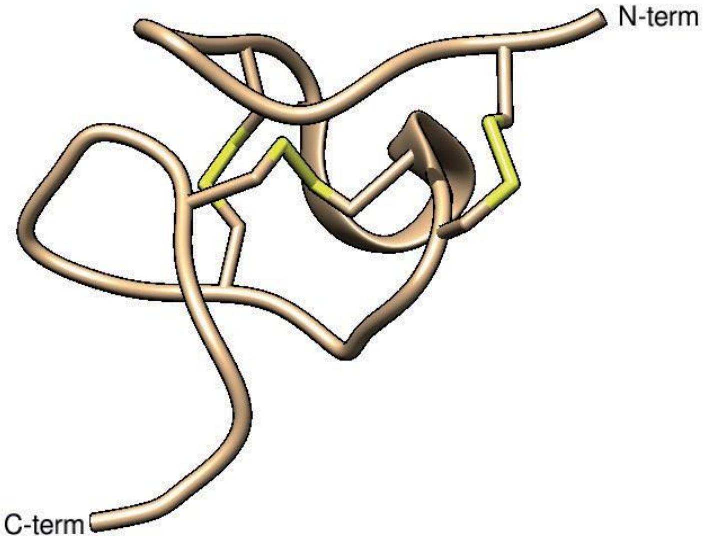

Figure S2. Nemertide alpha-1 3D ribbon structure with disulfide bonds coloured in yellow. PDB ID: 6ENA. [14].

Table S1. Molecular weight (g/mol) and monoisotopic mass (Da) of nemertide alpha-1.

Molecular Weight (g/mol)
3309.733

Monoisotopic Mass (Da)	
+0	3307.344
+1	3308.351
+2	1654.679
+3	1103.455
+4	827.8432
